# 13. Neurological manifestation in granulomatosis with polyangiitis

**DOI:** 10.1093/rap/rky033.005

**Published:** 2018-09-20

**Authors:** Geetha Lakshmi Janakiraman, Anupam Paul, Sri Nagarajan

**Affiliations:** 1 Rheumatology, James Cook University Hospital, Middlesbrough, UNITED KINGDOM; 2 Pathology, James Cook University Hospital, Middlesbrough, UNITED KINGDOM

**Introduction:** Granulomatosis with polyangiitis is rare disorder where there is inflammation of small blood vessels supplying the nose, throat, sinuses, lungs and kidneys. Peripheral and central nervous system manifestations are not uncommon occurring in 10-45% patients. Because of the non specific presenting symptoms, often there is a delay in diagnosis. Prompt diagnosis and treatment could prevent fatal complications.

**Case description:** A 47 year old journalist who was generally healthy, with recent onset of steroid responsive arthralgia was commenced on hydroxychloroquine. Two months later she presented with parieto occipital headache. Giant cell arteritis was suspected and high dose prednisolone 60 mg was commenced. Since the temporal artery biopsy was negative and a non contrast MRI head was normal, the prednisolone was quickly weaned down and stopped. Headaches progressively worsened over the next few weeks, and the patient was seen by neurology. She also had symptoms of blocked ears with ear pain with gradually progressing hearing loss over two months. Her symptoms evolved to have nasal bridge pain with yellow green blood stained nasal discharge not responding to courses of antibiotics for presumed sinusitis. She reported left sided facial numbness. Probing further connective tissue disease history she had history of blistering photosensitive rash, nausea, non-productive cough, night sweats and weight loss. Clinical examination revealed impaired pin prick sensation in the maxillary and mandibular division of the trigeminal nerve bilaterally but more pronounced on the right side. The Rinne’s test was indicative of conductive hearing loss with Weber’s test localising to the left. ENT examination concluded bilateral rhinitis and cobblestone appearance of right nasal septum with pustules. Other system examination was normal. Investigations revealed raised inflammatory markers ESR 71, CRP 160, neutrophilic leucocytosis, thrombocytosis with microcytic anaemia. Her proteinase 3 ANCA was positive at 9.4 with rest of the autoantibody screen being negative. She had trace of blood on urine analysis with normal urine protein creatinine ratio and kidney functions. A left lower lobe cavitation was noticed on chest x-ray, confirmed by CT scan to be a subpleural cavitating mass. There was bilateral multiple renal infarcts. Her MRI brain and CT sinuses was normal. The histopathology right nasal septal biopsy was consistent with Granulomatosis with polyangitis (GPA). She was promptly treated with three doses of intravenous methylprednisolone followed by six pulses of intravenous cyclophosphomaide. Her headaches persisted although less severe and her hearing loss was stable. She had a good serological response with PR3 ANCA levels reducing to 2.2 with normalisation of inflammatory markers. There was complete resolution of the chest x-ray nodule mirrored on CT scan. On reducing the prednisolone dose she had some recurrence of nasal crusting hence was commenced on oral methotrexate for maintenance of remission.

**Discussion:** Granulomatosis with polyangiitis could be difficult to diagnose as it presents with non specific symptoms. Increased awareness of the rare but fatal condition, appropriate investigations and prompt treatment would help with good recovery of patient. This would help to minimise morbidity and prevent mortality especially with neurological manifestation secondary to granulomatous inflammation.

**Key Learning Points:** Increased awareness, collating all seemingly non-specific symptoms and appropriate investigations will lead to early diagnosis of granulomatosis with polyangiitis (GPA). Multi-system involvement is not uncommon at presentation. Early liaison and close coordination among multiple specialties is therefore essential for the management of this condition. Nervous system involvement is less frequent than the classical presentation with lung and kidney involvement in GPA. Early treatment of granulomatous inflammation of the central nervous system helps with preventing and minimising neurological impairment and residual damage.

**Disclosure: G. Janakiraman:** None. **A. Paul:** None. **S. Nagarajan:** None.

